# Effects of Glyphosate and Roundup^®^ Herbicides on Cardiac and H9c2 Cells’ Mitochondrial Respiration and Oxidative Stress

**DOI:** 10.3390/ijms27104583

**Published:** 2026-05-20

**Authors:** Rayhana Rihani, Anne-Laure Charles, Walid Oulehri, Anne Lejay, Anne Charloux, Margherita Giannini, Alain Meyer, Bernard Geny

**Affiliations:** 1 Biomedicine Research Center of Strasbourg (CRBS), UR 3072, “Mitochondria, Oxidative Stress and Muscle Plasticity”, Faculty of Medicine, University of Strasbourg, 67000 Strasbourg, France; rayhana.rihani@etu.unistra.fr (R.R.);; 2 Department of Physiology and Functional Explorations, Faculty of Medicine, University Hospital of Strasbourg, 67091 Strasbourg, France; 3 Department of Anesthesiology, Faculty of Medicine, University of Strasbourg, 67000 Strasbourg, France; 4 Department of Vascular Surgery, University Hospital of Strasbourg, 67091 Strasbourg, France

**Keywords:** glyphosate, Roundup^®^, herbicide, mitochondria, mitochondrial respiration, hydrogen peroxide (H_2_O_2_), oxidative stress, heart

## Abstract

Herbicides, used worldwide to improve agricultural yields, are associated with pollution and significant health problems. Cardiac damage is a major concern, and the respective contributions of glyphosate (GP) and its commercial formulation, Roundup^®^ (RU), warrant investigation. We studied the specific effects of GP and RU on isolated rat cardiac mitochondria and on H9c2 cardiomyocytes cultured for 6 and 24 h to determine whether the potential cardiotoxicity of GP and/or RU are linked to impaired mitochondrial respiration and increased hydrogen peroxide (H_2_O_2_) production. To this end, we used various mitochondrial complex substrates and a high-resolution oxygraphy. Unlike the GP alone which demonstrated no significant effect, the RU decreased cardiac mitochondrial respiration (21.90 ± 2.99 vs. 41.23 ± 7.09 pmol/s/mL, −46.9%, *p* = 0.007) for OXPHOS CI in respectively the RU and the control groups. RU also impaired OXPHOS CI+II (−51.5%, *p* = 0.003), maximal mitochondrial respiration (ETS CI+II, −46.7%, *p* = 0.001) and coupling (−35.4%, *p* = 0.0003). Similarly, 24 h exposure to RU decreased H9c2 cell number (−48.59%, *p* = 0.0023) but increased their mitochondrial respiration (+38.2%, *p* = 0.03, +37.6%, *p* = 0.03, +43.2%, *p* = 0.03 for OXPHOS CI, OXPHOS CI+II and ETS CI+II respectively). We observed a similar trend (NS) after 24 h exposure to GP. In conclusion, these results support an enhanced cardiac toxicity of the Roundup^®^ as compared to the glyphosate. Both decreased mitochondrial respiration and increased hydrogen peroxide production were involved in isolated mitochondria impairment. After 24 h exposure to Roundup^®^, a compensatory mechanism potentially counterbalanced the decreased H9c2 cell number. These data support future studies aiming to reduce Roundup^®^-associated cardiac alterations not only by reducing its use but also by investigating the effectiveness of antioxidant and mitochondria-focused therapy.

## 1. Introduction

Glyphosate (GP, *N*-(phosphonomethyl)glycine) is an organophosphorus herbicide widely used to eliminate weeds. Over time, weeds have developed resistance, leading to an increase in GP doses and in the frequency of application in fields. Indeed, the inert carbon–phosphorus bond of GP makes it resistant to biodegradation and therefore allows longer persistence in the environment. GP inhibits the shikimate pathway, using the enzyme 5-enolpyruvylshikimate-3-phosphate synthase, present in plants, bacteria, fungi, and protists, but generally not found in humans or animals. Nevertheless, GP has been detected in animals and in humans, raising concerns about its safety, particularly given its widespread global use. Consistently, GP residues have been detected in environmental waters worldwide, including in drinking water [1,2,3,4,5,6,7].

Noteworthy, GP is generally associated with co-formulants like polyoxyethylene amine, which enhances the absorption and translocation of the active compound in plants. Co-formulants were previously considered as inactive components in terms of weed control but they clearly improve GP potency and unfortunately increase toxicity as compared to GP alone [8].

Thus, GP’s deleterious health effects are increased during exposure to other pollutants, and GP formulation with co-formulants appears worse than GP alone when considering human health [9,10]. The largely used Roundup^®^’s formulation (RU) demonstrated enhanced toxicity compared to GP alone, supporting adverse effects on non-target organisms [11]. Although controversial, many studies reported a deleterious effect of herbicides including GP and RU on different systems largely involved in homeostasis in animals and humans [8,12,13,14].

Concerning the cardiovascular system, both the vessels and the heart likely appeared as target organs of GP and RU^®^. In rats, GP induced slight vasodilation of intact aortas and inhibited muscle contraction in isolated atrium sections, leading to hypotension [15]. GP and RU^®^ have also been tested on cardiac organoids and disturbances in organoid beating were detected in agreement with studies confirming arrhythmia and conduction defect occurrences. More recently, electrocardiographic abnormalities were associated with acute GP toxicity in humans [16,17,18,19]. Further, a cardiotoxic effect of RU^®^ was reported, affecting cardiac contractility and leading to arrhythmias [20].

The mechanisms involved in such toxicity are not fully known, but besides calcium channel blockade, impaired mitochondrial function associated or not with increased oxidative stress might play a role. Indeed, GP increased reactive oxygen species (ROS) production and had cytotoxic and genotoxic effects, thereby damaging the neuroblastoma cells and hepatic kidney and adipose tissues due to either high ROS concentrations or insufficient antioxidants [21]. In SH-SY5Y neuroblastoma cells, for example, GP acted as a pro-oxidant [22]. A study on the nematode C. elegans showed that 24 h of exposure to the herbicide Touchdown, composed of 52.3% GP, inhibited mitochondrial respiration, disrupted the proton gradient, decreased ATP production, and increased H_2_O_2_ production [23]. Similarly, GBH Scout, containing 720 g/kg of GP, affected Danio rerio after one week of exposure, impairing mitochondrial respiratory chain function by inhibiting Complexes I and IV and inducing hyperpolarization of mitochondria in the central nervous system [24]. Exposure to GP increased the concentration of ROS in cultured human erythrocytes [25]. Accordingly, we recently reported that GP and RU^®^ induced mitochondrial dysfunction and increased ROS both in renal tissue and HK2 cells [11].

The aim of this study was therefore to investigate the effects of GP alone or in combination with surfactants present in the RU^®^ formulation, on the heart, specifically by determining mitochondrial respiration and oxidative stress production, both at the level of isolated mitochondria and at the cellular level. We hypothesized that both GP and RU^®^ could impair mitochondrial respiration and acutely increase hydrogen peroxide production in cardiac mitochondria as well as after 6 and 24 h in cultured H9c2 cells, a cardiomyocytes cell line.

## 2. Results

### 2.1. Effects of Glyphosate and Roundup^®^ Herbicides on Cardiac Isolated Mitochondria, Mitochondrial Respiration and Oxidative Stress

#### 2.1.1. Unlike Glyphosate Alone, the Roundup^®^ Significantly Impairs the Cardiac Mitochondrial Respiration

**Figure 1:**
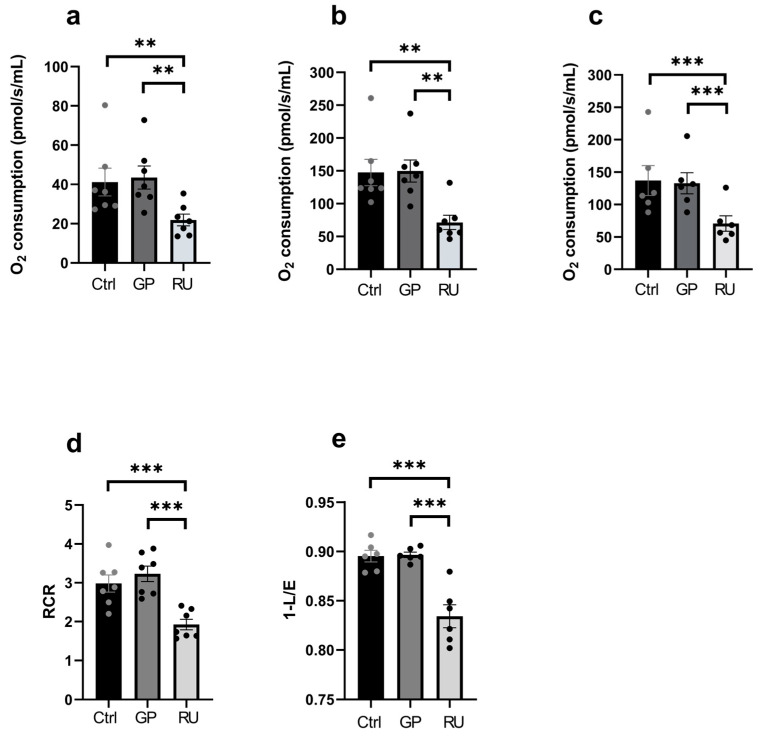
Effect of glyphosate and Roundup^®^ on mitochondria isolated from the heart following incubation of GP or RU for 30 min at 500 µM at 37 °C. (**a**) Oxygen consumption rate after addition of glutamate/malate and ADP substrates (OXPHOS CI). (**b**) Oxygen consumption rate after addition of glutamate/malate/ADP and succinate substrates (OXPHOS CI+II). (**c**) Oxygen consumption rate after titration by FCCP (ETS CI+II). (**d**) RCR, the ratio between OXPHOS CI and CI leak. (**e**) L/E ratio: L = Leak respiration and E = electron capacity transfer. ** *p* < 0.01; *** *p* < 0.001 Ctrl: control, GP: glyphosate, RU: Roundup^®^. RCR: Respiratory control ratio. *n* = 7 per group (except for Figure 1c where *n* = 6).

In the RU group, oxygen consumption in the OXPHOS CI state (oxidative phosphorylation supported by complexes CI, III, IV and V) was significantly reduced compared with the control group (−46.9%, *p* = 0.007) and compared to the GP group (−49.7%, *p* = 0.003), 41.23 ± 7.09, 43.52 ± 5.90, and 21.90 ± 2.99 pmol/s/mL, for control, GP and RU groups, respectively (Figure 1a). Similarly, OXPHOS CI+II (activate complex II to reach the maximum coupling state of OXPHOS) was significantly reduced in the RU group compared with the control group (−51.5%, *p* = 0.003) and compared to the GP group (−52.2%, *p* = 0.007), 147.5 ± 20.18, 149.8 ± 16.83, and 71.50 ± 10.87 pmol/s/mL for control, GP and RU group respectively (Figure 1b). At ETS CI+II (maximal electron transfer capacity), oxygen consumption was also significantly reduced in the RU group compared with the control group (−46.7%, *p* = 0.001) and compared to the GP group (−44.7%, *p* = 0.002), 119.5 ± 26.27, 115.1 ± 22.50, and 63.64 ± 12.30 pmol/s/mL for control, GP and RU groups, respectively (Figure 1c).

In contrast, we observed no significant variation in mitochondrial respiration in the GP group compared with the control group, whether in CI, CI+II, or ETS CI+II.

Finally, the coupling between oxidation and phosphorylation represented by the respiratory control ratio (RCR) showed a decrease in the group RU compared to the control group (−35.4%, *p* = 0.0003), and compared to the GP group (−40.4%, *p* = 0.0002), 2.98 ± 0.22, 3.23 ± 0.20, 1.93 ± 0.14 for control, GP and RU groups, respectively (Figure 1d). As suggested by Gnaiger et al., we also calculated the 1-L/E ratio, proposed to investigate mitochondrial coupling and potentially replacing the RCR [26]. Again, the 1-L/E ratio was decreased in the group RU compared to the control and the GP groups, 0.895 ± 0.005, 0.896 ± 0.002, and 0.834 ± 0.011 for control, GP and RU groups, respectively, *p* < 0.001. (Figure 1e).

#### 2.1.2. Unlike Glyphosate Alone, the Roundup^®^ Significantly Increases Cardiac Mitochondrial Hydrogen Peroxide Production

**Figure 2:**
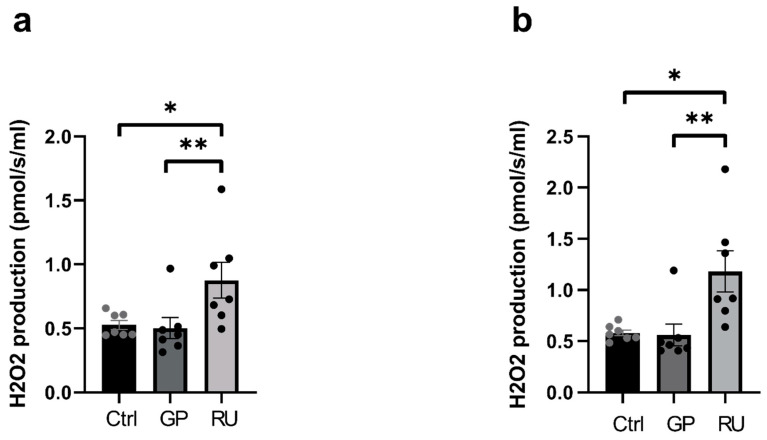
Effect of glyphosate and Roundup^®^ on H_2_O_2_ production in mitochondria isolated from the heart following incubation of GP or RU for 30 min at 500 µM at 37 °C. (**a**) H_2_O_2_ production rate after addition of glutamate/malate and ADP substrates (OXPHOS CI). (**b**) H_2_O_2_ production rate after addition of glutamate/malate/ADP and succinate substrates (OXPHOS CI+CII) * *p* < 0.05; ** *p* < 0.01. Ctrl: control, GP: glyphosate, RU: Roundup^®^. *n* = 7 per group.

In the RU group, analyzing OXPHOS CI, the H_2_O_2_ level increased significantly compared to the control group (+66.2%, *p* = 0.02) as well as to the GP group (+74.75%, *p* = 0.001), 0.53 ± 0.03, 0.50 ± 0.08 and 0.88 ± 0.14 pmol/s/mL for control, GP and RU groups, respectively (Figure 2a). There was no effect when comparing the GP to the control group.

When complexes I and II were activated (OXPHOS CI+II), similar results were obtained. In the RU group, H_2_O_2_ production increased significantly compared to the control group (+103.2%, *p* = 0.02) and compared to the GP group (+110.8%, *p* = 0.001), 0.58 ± 0.03, 0.56 ± 0.11 and 1.18 ± 0.20 pmol/s/mL for control, GP and RU groups, respectively (Figure 2b).

### 2.2. Effects of Glyphosate and Roundup^®^ Herbicides on Cardiac Cells H9c2

#### 2.2.1. Effects of 6 Hour Exposure to Glyphosate Alone or Roundup^®^ on H9c2 Cell Count, Mitochondrial Respiration and Hydrogen Peroxide Production

A. Neither glyphosate nor Roundup^®^ affect H9c2 cell number after 6 h exposure

**Figure 3:**
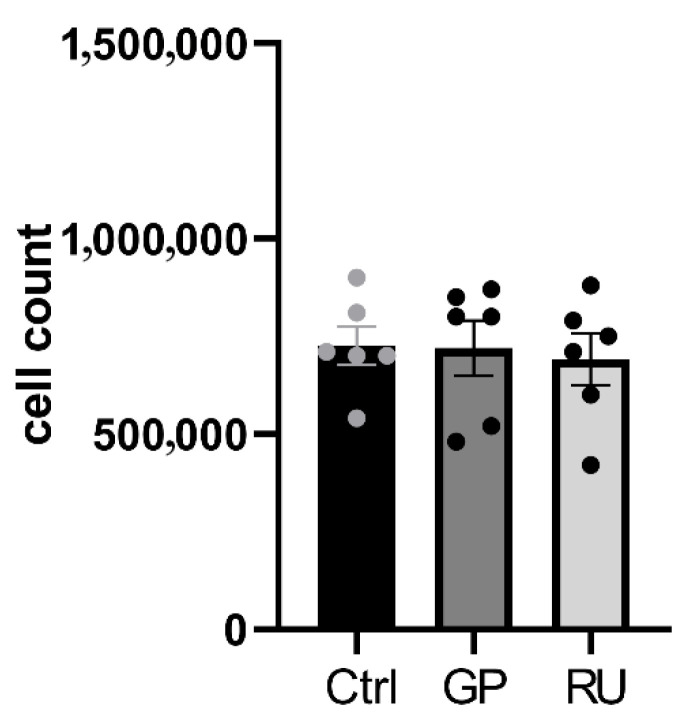
Effect of glyphosate and Roundup^®^ on H9c2 cell count after 6 h of exposure to 500 μM at 37 °C. Ctrl: control, GP: glyphosate, RU: Roundup^®^. *n* = 6 per group.

After 6 h of exposure, no significant difference in H9c2 cell population was observed between the glyphosate-treated and Roundup^®^-treated cells compared to the control group (7.3 × 10^5^ ± 0.49 × 10^5^, 7.2 × 10^5^ ± 0.7 × 10^5^, and 6.9 × 10^5^ ± 0.7 × 10^5^ for control, GP, and RU groups, respectively, Figure 3).

B. Neither glyphosate nor Roundup^®^ affect H9c2 cell mitochondrial respiration after 6 h exposure

**Figure 4:**
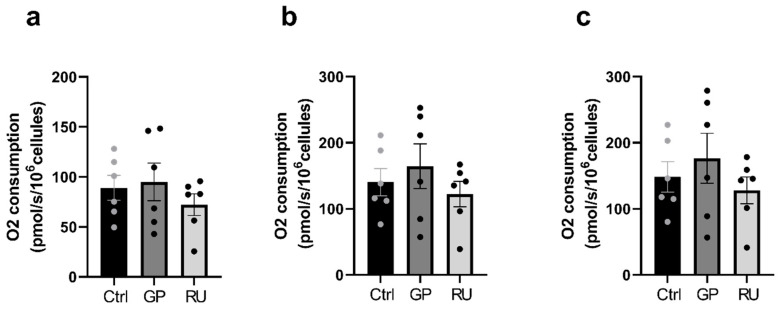
Effect of 6 h exposure to glyphosate and Roundup^®^ on oxygen consumption in H9c2 cardiac cells. (**a**) Oxygen consumption rate after ADP addition (OXPHOS CI). (**b**) Oxygen consumption rate after succinate addition (OXPHOS CI+CII). (**c**) Oxygen consumption rate after FCCP titration (ETS CI+CII). GP: glyphosate, RU: Roundup^®^, OXPHOS: oxidative phosphorylation, ETS: electron transfer capacity. *n* = 6 per group.

When only complex I was activated following ADP injection, no significant difference in oxygen consumption was observed between cells exposed to glyphosate or Roundup^®^. At the CI+II stage, after Complex II activation by succinate, there was also no significant difference between the groups. After successive FCCP titration to determine the maximal electron transfer capacity (ETS CI+II), no significant differences in oxygen consumption were observed between the groups (Figure 4).

C. Neither glyphosate nor Roundup^®^ affect H9c2 cell H_2_O_2_ production after 6 h exposure

H_2_O_2_ production was determined under the same conditions as oxygen consumption, using the same cells and in parallel with substrate addition. H_2_O_2_ production was measured by fluorimetry using the respirometer, based on the reaction between Amplex Red and H_2_O_2_.

**Figure 5:**
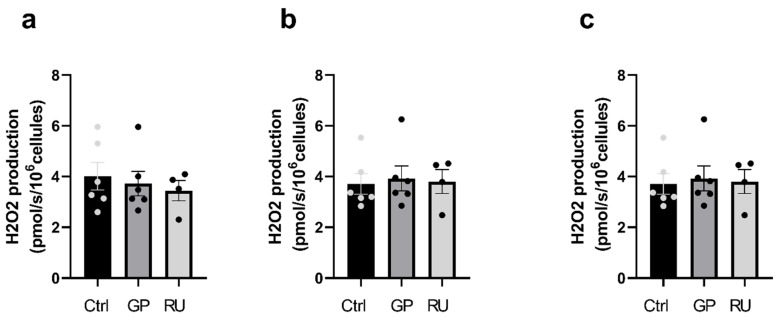
Effect of 6 h exposure to glyphosate and Roundup^®^ on H_2_O_2_ production of H9c2 cardiac cells. (**a**) Rate of H_2_O_2_ production after ADP addition (CI). (**b**) Rate of H_2_O_2_ production after succinate addition (CI+CII). (**c**) Rate of H_2_O_2_ production after FCCP titration (ETS CI+II). Ctrl: control, GP: glyphosate, RU: Roundup^®^. *n* = 6 per group.

After 6 h of exposure to glyphosate or Roundup^®^, no significant differences in H_2_O_2_ production were observed at the CI, CI+II, or ETS CI+II stages (Figure 5).

#### 2.2.2. Effects of 24 h Exposure to Glyphosate Alone or Roundup^®^ on H9c2 Cell Count, Mitochondrial Respiration and Hydrogen Peroxide Production

A. Roundup^®^ but not glyphosate significantly decreased H9c2 cell number after 24 h exposure

**Figure 6:**
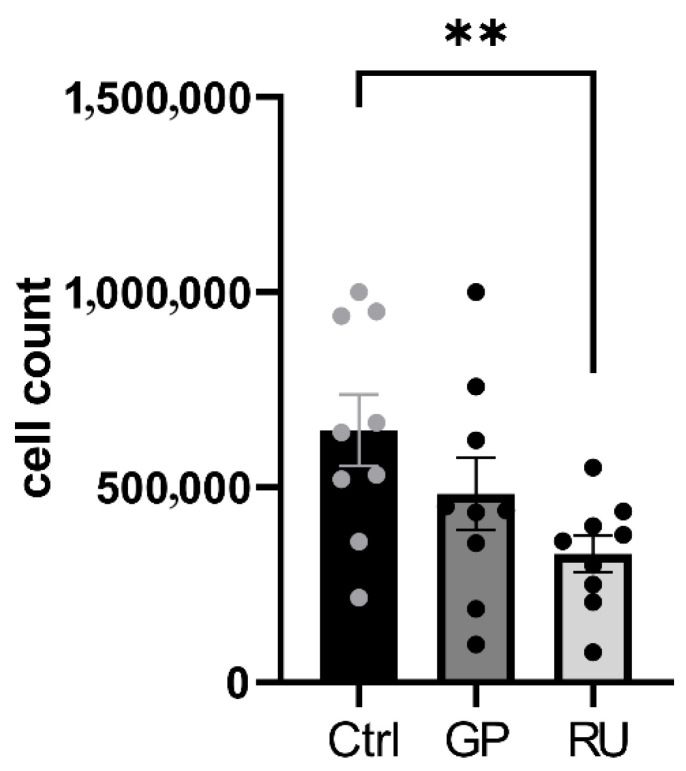
Effect of glyphosate and Roundup^®^ on H9c2 cell count after 24 h of exposure to 500 μM at 37 °C. **: *p* < 0.01; Ctrl: control, GP: glyphosate, RU: Roundup^®^. *n* = 9 per group.

After 24 h exposure, a significant decrease (−48.59%) in cell count was observed between the control group and the Roundup^®^ group (6.5 × 10^5^ ± 0.9 × 10^5^ and 3.3 × 10^5^ ± 0.5 × 10^5^, *p* = 0.0023). Regarding the glyphosate group, the trend to decrease was not significant compared to the control group (4.8 × 10^5^ ± 0.9 × 10^5^, −31.8% *p* = 0.08). Thus, the RU was more deleterious than the GP (Figure 6).

B. Roundup^®^ but not glyphosate significantly increased H9c2 cell mitochondrial respiration after 24 h exposure

**Figure 7:**
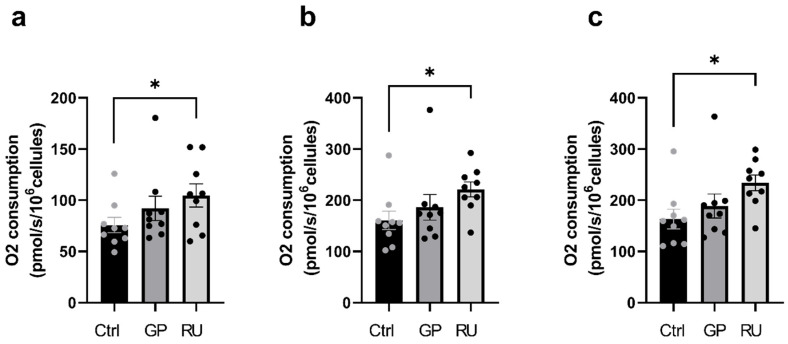
Effect of 24 h exposure to glyphosate and Roundup^®^ on oxygen consumption in H9C2 cardiac cells. (**a**) Oxygen consumption rate after ADP addition (OXPHOS CI). (**b**) Oxygen consumption rate after succinate addition (OXPHOS CI+CII). (**c**) Oxygen consumption rate after FCCP titration (ETS CI+CII). * *p* < 0.05, Ctrl: control, GP: glyphosate, RU: Roundup^®^. *n* = 9 per group.

In the RU group, oxygen consumption in the OXPHOS CI state was significantly increased compared with the control group (+38.2%, *p* = 0.03) 75.74 ± 7.56 and 104.7 ± 11.35 pmol/s/mL), (Figure 7a). Similarly, the OXPHOS CI+II respiration was significantly increased in the RU group compared with the control group (+37.6%, *p* = 0.03) 160.7 ± 18.26, and 221.1 ± 14.68 pmol/s/mL (Figure 7b). At ETS CI+II, oxygen consumption was also significantly increased in the RU group compared with the control group (+43.2%, *p* = 0.03) 163.3 ± 19.02, and 233.9 ± 15.41 pmol/s/mL (Figure 7c).

In contrast, no significant variation in mitochondrial respiration was observed in the GP group compared with the control group, whether in CI, CI+II, or ETS CI+II sates.

C. Neither glyphosate nor Roundup^®^ affect H9c2 cell H_2_O_2_ production after 24 h exposure

**Figure 8:**
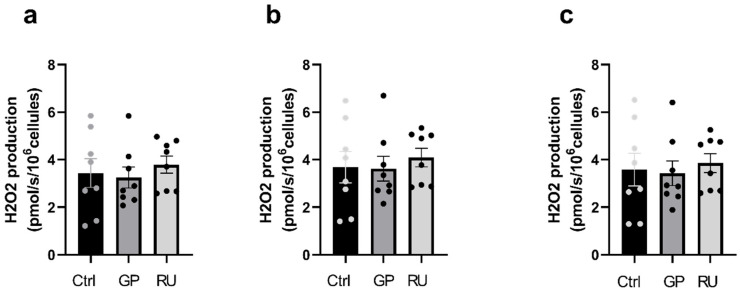
Effect of 24 h exposure to glyphosate and Roundup^®^ on H_2_O_2_ production of H9c2 cardiac cells. (**a**) Rate of H_2_O_2_ production after ADP addition (CI). (**b**) Rate of H_2_O_2_ production after succinate addition (CI+CII). (**c**) Oxygen consumption rate after FCCP titration (ETS CI+CII). Ctrl: control, GP: glyphosate, RU: Roundup^®^. *n* = 9 per group.

After 24 h exposure, H_2_O_2_ production on cardiac cells was not modified with glyphosate alone nor with Roundup^®^ (Figure 8).

## 3. Discussion

The main results of this study show that, unlike glyphosate, Roundup^®^ significantly decreased isolated cardiac mitochondrial respiration through complex I and II and decreased mitochondrial coupling. Roundup^®^ also increased hydrogen peroxide production. Furthermore, the investigations performed on cardiac cell culture demonstrate no change after 6 h considering cell number, mitochondrial respiration, or H_2_O_2_ production. Interestingly, we observed a significant decrease in H9c2 cell number after 24 h exposure to Roundup^®^, associated with a significant increase in mitochondrial respiration (OXPHOS CI, OXPHOS CI+CII, and ETS CI+CII). A similar trend was observed after 24 h exposure to GP.

### 3.1. Unlike Glyphosate Alone, Roundup^®^ Acutely and Significantly Impairs Mitochondrial Respiration and Increases Hydrogen Peroxide Production in Isolated Cardiac Mitochondria

Glyphosate alone showed no significant effects on isolated cardiac mitochondrial respiration and hydrogen production. These data are in line with other reports, demonstrating few deleterious effects of GP alone compared to formulations containing surfactants [27]. Peixoto et al. also demonstrated a greater toxic effect of RU than GP on isolated liver mitochondria [28]. Furthermore, Kim et al., observed that glyphosate alone had no effect, but POEA combined with glyphosate had a synergistic effect, inducing apoptosis in H9c2 cells. The authors suggested that POEA altered the toxicodynamic of glyphosate, damaging the cell membrane and facilitating glyphosate entry, inducing its toxic effects [29]. The study by Printemps et al. supports this hypothesis, as they described in human cardiomyocytes that Roundup^®^ blocked calcium channels leading to severe cardiac toxicity with reduced contractility and enhanced arrhythmias. At an equal concentration of glyphosate alone, no alteration was observed [20].

Concerning oxidative stress, the Roundup^®^-induced impairment in mitochondrial respiration was associated with an increase in hydrogen peroxide production. Such mitochondrial enhancement in ROS production is consistent with previous data. In porcine oocytes exposed for 44 h to Roundup, ROS production significantly increased. When the same experiment was performed with glyphosate alone, no significant increase in ROS was observed, suggesting that Roundup mediates glyphosate’s toxic effect [30]. Accordingly, GP-surfactantbased exposure significantly elevated mitochondrial H_2_O_2_ levels compared to control and GP-treated cells [31]. Furthermore, in the fish Odonthesthes humensis, a decrease in NADH dehydrogenase 2 was observed after 24 h of exposure to Roundup. This gene alteration likely impaired mitochondria, electron transport, and increased ROS production. The study suggests that apoptosis may be triggered by cell membrane damage or mitochondrial ROS production [32].

Interestingly, Mesnage et al. used a wide “omic” approach allowing determine changes in the epigenome (DNA methylation profile), transcriptome, and miRNA profiles in the liver of rats exposed to glyphosate or Roundup MON 52276. Roundup induced oxidative stress and an unfolded protein response at concentrations at which glyphosate had no effects [33].

### 3.2. Twenty-Four Hours Exposure to Roundup^®^ Significantly Decreased H9c2 Cell Number and Increased Mitochondrial Respiration

To go further, we compared the effects of GP and RU on H9c2 cardiomyocyte cell lines. No significant change was observed after 6 h exposure, suggesting that these cells might be more resistant to the herbicides than isolated mitochondria. Indeed, isolated mitochondria (outside their protective environment) might be more prone to damage than mitochondria located in cells. Accordingly, comparison of homogenates with isolated mitochondria prepared from ventricular myocardium demonstrated a better preservation of mitochondrial membranes and interestingly, mitochondria with an intact outer membrane exhibited greater protective effects against oxidative damage than those with damaged outer membranes, particularly in terms of enhancing cell survival under severe oxidative stress conditions [34,35]. Additionally, the oxidative characteristics of striated muscles is known to be protective when submitted to injuries [36].

On the other hand, after 24 h exposure to Roundup^®^, H9c2 cell number significantly decreased. GP only tended to induce such deleterious effects [37]. Apoptosis and/or cell cycle blockade might have played a role. Indeed, Kim et al. observed in rat H9c2 cells a significant decrease in BCL-2 (anti-apoptotic) and an increase in BAX (pro-apoptotic) expression, associated with a release of cytochrome C and increased caspases activity [29]. Similarly, apoptosis was clustered in the heart and vascular regions and apoptosis-related genes including caspase-3, caspase-9, Bax, and bcl-2 were altered in zebra-fish subjected to GP [38]. Recent data further support the involvement of the apoptosis pathway in GP-associated reduction in cardiac myoblast viability [39].

Besides apoptosis, inhibition of the cell cycle might also participate in the reduced H9c2 cell number observed after RU exposure in our study. Indeed, pesticides can affect cell viability by inducing cell cycle arrest likely stopping cell division, growth and proliferation of cardiac cell line H9c2 [40].

Interestingly and somewhat unexpectedly, we observed an enhanced mitochondrial respiration 24 h after exposure to RU. Rather than showing a lack of deleterious effects of the herbicides, this might correspond to a compensatory mechanism aiming to reduce the decreased cell viability. Accordingly, Arrigo et al. observed a complete restauration of H9c2 cell phenotype 24 h after GP exposure which suggests that these cells can recover promptly after damage [39]. Thus, mitochondrial stress can result in local beneficial outcomes within the tissue or cell type experiencing that stress, likely through mitohormesis leading to enhanced mitochondrial number and/or function [41,42]. Further work is nevertheless needed to determine the mechanisms beyond the increased mitochondrial respiration rate despite reduced cell number after 24 h exposure to RU, particularly investigating potential reversibility of cell alteration such as decrease in ATP content and/or oxidative stress-induced damages. Indeed, the switch from mitochondrial metabolic shutdown to mitochondrial biogenesis is finely orchestrated by many factors [43,44].

The clinical relevance of these herbicide-induced injuries was not investigated in our study. However cardiac mitochondrial dysfunction and increased oxidative stress are known to be related to chronic or acute cardiac failure [45,46]. More specifically, although clearer causal relationship still needs to be proven, cardiac toxicity of GP and RU have been proposed in humans. Thus, reports showed electrocardiographic abnormalities and/or reduced systemic blood pressure after GP and/or RU exposure [19,47,48,49]. Furthermore, Moon at al. observed an association between PR prolongation and fatality mainly in glyphosate isopropyl amine salt herbicide poisoning cases [50].

Limitations of the study.

The results are limited to the specific formulation used and cannot be extrapolated to other experimental conditions. It can be difficult for scientists to elucidate the impacts of the various co-formulants in commercial RU, as their precise composition is often confidential. Greater transparency would be beneficial in the future [51,52,53]. Nevertheless, further studies will be useful to better determine the effect caused by the co-formulant and particularly whether the co-formulant alone is cardiotoxic and/or facilitates glyphosate cellular entry, enabling glyphosate-mediated toxicity or whether the co-formulant and glyphosate act synergistically through distinct mechanisms [29]. Our approach might also have been enhanced by evaluating dose-response relationships but the doses analyzed remain relevant in light of previous studies [11,54,55].

Furthermore, the mechanisms underlying the increase in mitochondrial respiration after 24 h of RU exposure warrant further investigation, as it is unknown whether this results from more robust mitochondria in the surviving cell population, reflecting differential selection rather than compensation. In this view, determining the reversibility of change in ATP content or oxidative damage potentially participating in RU-induced cell number decrease will be interesting in future studies.

## 4. Materials and Methods

### 4.1. Study Design

**Figure 9:**
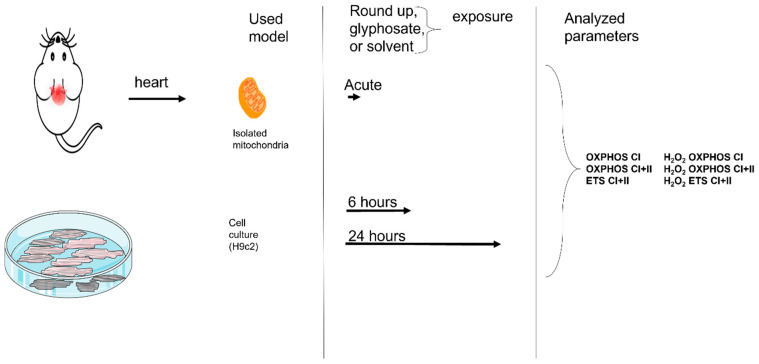
Study design.

These investigations were performed on mitochondria isolated from rat’s heart and on cultured H9c2 cell lines (Figure 9).

#### 4.1.1. Experimental Design

Eleven five-month-old male Wistar rats, obtained from Janvier Labs (Le Genest-St-Isle, France), were included in this study. Housed in an enriched environment maintained at 22 ± 2 °C with a 12-h light/dark cycle, the animals had free access to food and water. Anesthesia was induced and maintained using 4% isoflurane in oxygen via an induction chamber. All animals were euthanized prior to heart collection. Tissues intended for mitochondrial isolation were immediately placed in a cold isolation buffer (250 mM sucrose, 10 mM Tris/HCl, 0.1 mM EGTA, pH 7.4) maintained at 4 °C.

#### 4.1.2. Solutions Used

The glyphosate [*N*-(phosphonomethyl) glycine] (100% purity, Pestanal^®^ (MilliporeSigma, Burlington, MA, USA)) was prepared at a starting concentration of 12 g/L, dissolved in distilled water and then heated to 37 °C under ultrasound for 15 min. The Roundup^®^ (ROUNDUP++ UFI KPF1-E0Y7-K009-RH2Q Code6789574, Bayer CropScience SA-NV J.E. Mommaertslaan 14 1831 Diegem (Machelen, Belgium) was similarly diluted 1:30 to contain 12 g/L of glyphosate from a stock solution at 360 g/L. Isolated cardiac mitochondria were exposed to a final glyphosate concentration of 500 μM (85 mg/L), either in its pure form or as a part of the Roundup^®^ formulation as used previously in the kidney study [11]. Indeed, GP concentration in blood was 61 mg/L (0.6 to 150 mg/L) in mild and moderate intoxications and when lethal, GP concentrations reached 690 to 7480 mg/L [54,55]. It is worth noting that the concentration of GP was four times higher in human seminal plasma than in blood plasma [49]. Although the concentrations of GP in the heart itself remain unknown, an increased concentration could also be observed in this organ with high perfusion values.

### 4.2. Cardiac Mitochondria Isolation

Briefly, mitochondria were obtained using a stepwise differential centrifugation process. Heart was cut in small pieces and then transferred to gentleMACS™ C tubes adapted to the gentleMACS dissociation grinding device (Miltenyi Biotec, Teterow, Germany). After the series of differential centrifugations, a pellet of concentrated mitochondria was obtained. Then, protein concentration was measured using a Bradford assay to estimate the mitochondrial population. The amount of 0.8 mg was exposed for 30 min to glyphosate 500 µM (GP) or the commercial herbicide Roundup composed by glyphosate (500 µM) and surfactants (RU), and compared to a control group exposed to the solvent in the same concentrations as the other groups.

### 4.3. H9c2 Cell Culture

#### 4.3.1. Cell Proliferation

H9c2 cells are derived from rat embryonic cardiomyocytes, produced by ATCC. They are used as an alternative cardiomyocyte model to study cardiac pathologies. Cells were cultured in Dulbecco’s Modified Eagle Medium (DMEM, Thermofisher, Waltham, MA, USA), with 1 g/L glucose, supplemented with 10% fetal bovine serum and gentamicin (antibiotic), and incubated at 37 °C in a 5% CO_2_ atmosphere. Cells were maintained below 70–80% confluence.

#### 4.3.2. Differentiation of H9c2 Cells

For the experiments, H9c2 cells were differentiated into cardiomyocytes using retinoic acid (1 nM), allowing us to obtain cardiac cells rather than skeletal muscle cells. Differentiation requires 5 to 7 days to reach maximum efficiency.

#### 4.3.3. Cell Treatment

Differentiated cells were then divided into three groups. The control group (CTRL) was exposed for 6 or 24 h to the solvent of the two herbicides (water). The second group was exposed for 6 or 24 h to glyphosate (500 µM), glyphosate group (GP). The third group was exposed for 6 or 24 h to the commercial herbicide Roundup composed by glyphosate (500 µM) and surfactants, roundup group (RU). Both GP and RU were incubated in culture flasks in a buffered solution with a 1:149 ratio.

### 4.4. Mitochondrial Respiration

The mitochondrial respiration was investigated using a high-resolution respirometer (Oxygraph-2k, Oroboros Instruments, Innsbruck, Austria). Measurements were determined with continuous stirring at 37 °C. The measurement was performed in 2 mL of MiR05 (EGTA 0.5 mM, MgCl2 3 mM, K lactobionate 60 mM, Taurine 20 mM, KH_2_PO_4_ 10 mM, HEPES 20 mM, Sucrose 110 mM, BSA 1 mg/mL, Creatine 20 mM). The Oxygraph is equipped with two Clark electrodes (oxygen sensitive electrodes), each connected to an Oxygraph chamber. Once basal respiration stabilized, a titration protocol using different substrates and inhibitors was applied to analyze the oxidative phosphorylation. First, glutamate (5 mM) and malate (2 mM) were added to assess non-phosphorylating respiration initially supported by complex I. Then, addition of ADP (2 mM) activated complex V (ATP synthase), revealing the oxidative phosphorylation, supported by complexes CI, III, IV and V (OXPHOS CI). Succinate (25 mM) was then injected to activate complex II and reach the maximum coupling state of OXPHOS (OXPHOS CI+II), as previously reported [56].

To assess the maximal electron transfer capacity within the mitochondrial respiratory chain, FCCP (0,25 µM) was added by successive titration (ETS CI+II). Results are expressed as pmol/s/mL.

Mitochondrial coupling was assessed using the respiratory control ratio (RCR). Although already published value was below 4 [34], this might indicate compromised coupling, and we therefore additionally investigated mitochondrial coupling by determining the 1-L/E ratio.

### 4.5. H_2_O_2_ Production

Hydrogen peroxide (H_2_O_2_) production in response to the sequential addition of substrates and inhibitors was studied simultaneously with O_2_ consumption using a high-resolution respirometer (Oxygraph-2k, Oroboros Instruments, Innsbruck, Austria) on isolated mitochondria and cell culture.

H_2_O_2_ production was measured using Amplex Red reagent (20 µM, Invitrogen, Carlsbad, CA, USA), which reacts stoichiometrically (1:1) with hydrogen peroxide in a reaction catalyzed by HRP (1 U/mL horseradish peroxidase; Fluka Biochemika, Darmstadt, Germany) to give the fluorescent compound resorufin. Resorufin has excitation/emission wavelengths of 563/587 nm and is highly stable once formed [57].

### 4.6. Statistical Analysis

Statistical analysis was performed using GraphPad Prism 8 (v8.4.3 GraphPad Software, Inc., San Diego, CA, USA). Results are presented as mean ± SEM (Standard Error of the Mean), with “n” indicating the number of samples. The Shapiro–Wilk test was used to determine if the values followed a normality curve. For a normal distribution, comparisons between control, glyphosate, and Roundup^®^ groups were conducted using an ANOVA test, followed by a Fisher’s LSD post hoc test. If the data did not follow a normal distribution, a non-parametric Friedman test was performed. The significance threshold for the *p*-value was set at 0.05.

## 5. Conclusions

In conclusion, this study highlights the effects of Roundup^®^ and glyphosate on cardiac mitochondria and H9c2 cardiomyocyte cell lines. Results support an enhanced cardiac toxicity of the Roundup^®^ as compared to the glyphosate. A decrease in mitochondrial respiration and an increase in hydrogen peroxide production in isolated mitochondria participate in the acute deleterious effects of herbicide. After 24 h exposure to Roundup^®^, we observed an enhanced mitochondrial respiration, potentially counterbalancing the decreased cell number. These data support future studies aiming to reduce Roundup^®^-associated cardiac alterations not only by reducing its use but also by investigating the usefulness of antioxidant and mitochondria-focused therapy.

## Data Availability

The original contributions presented in this study are included in the article. Further inquiries can be directed to the corresponding author.
